# A Clustering Approach to Meal-Based Analysis of Dietary Intakes Applied to Population and Individual Data

**DOI:** 10.1093/jn/nxac151

**Published:** 2022-07-11

**Authors:** Cathal O'Hara, Aifric O'Sullivan, Eileen R Gibney

**Affiliations:** Insight Centre for Data Analytics, University College Dublin, Dublin, Ireland; UCD Institute of Food and Health, University College Dublin, Dublin, Ireland; School of Agriculture and Food Science, University College Dublin, Dublin, Ireland; UCD Institute of Food and Health, University College Dublin, Dublin, Ireland; School of Agriculture and Food Science, University College Dublin, Dublin, Ireland; Insight Centre for Data Analytics, University College Dublin, Dublin, Ireland; UCD Institute of Food and Health, University College Dublin, Dublin, Ireland; School of Agriculture and Food Science, University College Dublin, Dublin, Ireland

**Keywords:** dietary assessment, generic meals, meal patterns, clustering, food combinations

## Abstract

**Background:**

Examination of meal intakes can elucidate the role of individual meals or meal patterns in health not evident by examining nutrient and food intakes. To date, meal-based research has been limited to focus on population rather than individual intakes, without considering portions or nutrient content when characterizing meals.

**Objectives:**

We aimed to characterize meals commonly consumed, incorporating portions and nutritional content, and to determine the accuracy of nutrient intake estimates using these meals at both population and individual levels.

**Methods:**

The 2008–2010 Irish National Adult Nutrition Survey (NANS) data were used. A total of 1500 participants, with a mean ± SD age of 44.5 ± 17.0 y and BMI of 27.1 ± 5.0 kg/m^2^, recorded their intake using a 4-d weighed food diary. Food groups were identified using *k*-means clustering. Partitioning around the medoids clustering was used to categorize similar meals into groups (generic meals) based on their Nutrient Rich Foods Index (NRF9.3) score and the food groups that they contained. The nutrient content for each generic meal was defined as the mean content of the grouped meals. Seven standard portion sizes were defined for each generic meal. Mean daily nutrient intakes were estimated using the original and the generic data.

**Results:**

The 27,336 meals consumed were aggregated to 63 generic meals. Effect sizes from the comparisons of mean daily nutrient intakes (from the original compared with generic meals) were negligible or small, with *P* values ranging from <0.001 to 0.941. When participants were classified according to nutrient-based guidelines (high, adequate, or low), the proportion of individuals who were classified into the same category ranged from 55.3% to 91.5%.

**Conclusions:**

A generic meal–based method can estimate nutrient intakes based on meal rather than food intake at the sample population and individual levels. Future work will focus on incorporating this concept into a meal-based dietary intake assessment tool.

## Introduction

Because nutrients and foods are consumed as part of meals, meal-based intake information may complement existing dietary guidelines with regard to understanding and meal planning by consumers (1, 2). Knowledge derived from meal-based research would allow dietary guidelines to provide advice on the basis of different meal types and their combinations, the timing of meals, and the types of foods consumed together as part of those meals, rather than focusing only on the types of foods that should be consumed over the course of a day (2, 3). This advice may be more intuitively understood because people tend to conceptualize their dietary intakes as meals rather than daily intakes of foods (4, 5). A meal-based approach may also be superior in technology-based intake assessments and the provision of personalized nutrition recommendations, because meal intakes can be recorded in a way that is less burdensome than recording of individual food intakes (6).

Previous meal-based research has used national dietary survey data to identify a small list of generic, or commonly consumed, meals in a given population that are representative of the much larger list of actual meals consumed by that population (7–11). These generic meals typify the actual meals that are consumed and are defined by their nutritional content and the food groups that they contain (7–11). Research on generic meals to date has focused on their potential for estimating dietary intakes at a population level (7–9) or characterizing the types of foods that individuals commonly combine to make meals (7–11). No studies have assessed whether generic meals can be used to classify individuals’ dietary intakes into categories according to nutrient-based dietary guidelines. This method of categorizing individual intakes has been used in other forms of technology-based personalized nutrition, whereby the dietary advice provided to individuals is determined by whether they consume low, moderate, or high quantities of nutrients according to nutrient-based guidelines (12). The use of technology in dietary intake assessment can reduce respondent burden (13), and meal-based methods have been proposed as a lower-burden way in which dietary intakes could be assessed with regard to nutrient-based guidelines and dietary feedback provided using Internet and mobile technology (6). Determining the ability of meal-based intakes to correctly estimate nutrient intakes at an individual level is important for the development of such meal-based intake assessment and feedback tools.

Good agreement with food-based methods (such as food diaries) has been obtained using these generic meals to determine mean daily intakes at a sample population level, with median correlation coefficients ranging from 0.44 (7) to 0.61 (9) for correlations between estimated nutrient intakes derived from the food data and the generic data. However, methods of identifying generic meals to date have failed to incorporate portion sizes and have limited emphasis on nutritional content when identifying generic meals. This may limit their ability to capture the variability of nutrient intakes between individuals and may not be sufficient to identify generic meals and meal patterns that are relevant for the promotion of health (7, 8, 14). The approaches used to date have relied on the frequent itemset data mining methods (7–9), latent variable mixture modeling (10), and principal components analysis (11). Although these approaches are effective in identifying common combinations of food groups within meals, they cannot distinguish between those meals that contain similar or identical food groups but are considerably different in their nutritional contents, for example, the foods in the meat/fish group can vary considerably with regard to their energy content and fat composition (7, 9–11, 15).

The objectives of this study are to determine generic meals in national dietary survey data incorporating both portion size and the nutritional content of meals, and to assess the degree of agreement between nutrient intakes derived using the generic meals and the original meals at both the sample population and individual levels.

## Methods

### Study sample

The analysis was carried out on previously collected data from the National Adult Nutrition Survey (NANS) in Ireland (16). NANS recruited participants aged between 18 and 90 y who were free-living and not pregnant or breastfeeding. Data were collected between October 2008 and April 2010. The sample was representative of Irish adults with respect to age, gender, social class, and geographical location. Each participant completed a 4-d weighed food diary. Reported food intake data were converted into nutrient data using WISP© (Tinuviel Software). In this original data set each participant had nutrient intake values for each food consumed during the recording period. Ethical approval was provided by University College Cork Clinical Research Ethics Committee of the Cork Teaching Hospitals. Further details on the study sample are available elsewhere (16).

### Food groups

**FIGURE 1:**
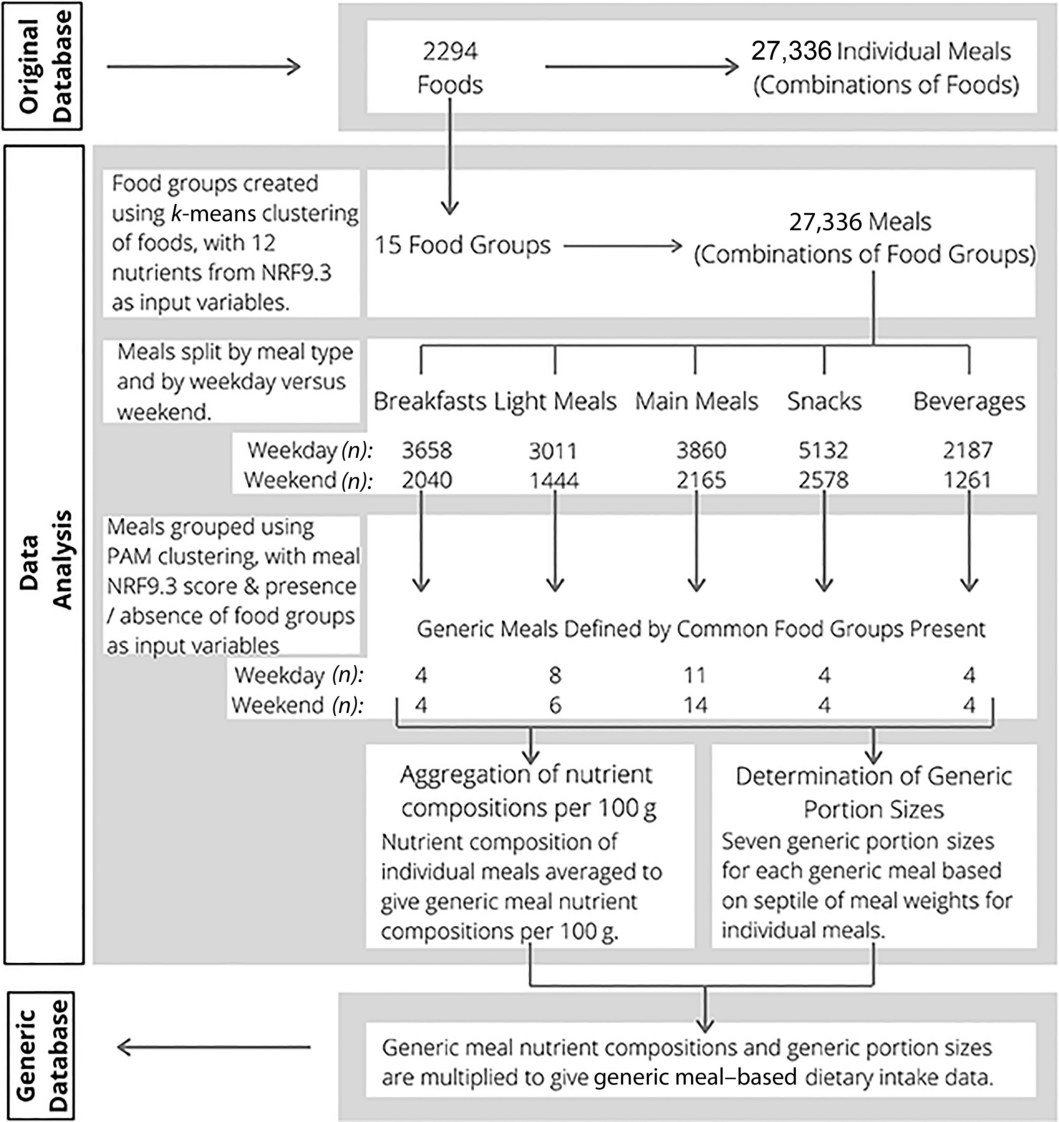
Flowchart of the process for deriving generic meals. The original database is from 1500 participants recording their dietary intakes over a period of 4 d during the National Adult Nutrition Survey in Ireland, 2008–2010. NRF9.3, Nutrient Rich Foods Index; PAM, partitioning around the medoids.

Clustering is a data analysis technique that groups observations (in this case, foods) into groups, or clusters, in such a way that the features (or variables; in this case, the nutrients of interest within the foods) of the observations within a given cluster are more similar to each other than to the observations (foods) in other clusters (17). Within the current analysis, foods reported in the dietary survey were firstly grouped into 1 of 6 groups within the Irish Food pyramid (vegetables, salad, and fruit; cereal, bread, potato, pasta, and rice; milk, yogurt, and cheese; meat, poultry, fish, eggs, beans, and nuts; fats, spreads, and oils; and foods high in fat, sugar, and/or salt) (18), and then further grouped using *k-*means clustering (**Figure 1**). A seventh group (other) was used for the remaining foods that did not belong to any of the aforementioned groups. This approach thus identified subgroups of foods within each of the existing food pyramid food groups, with similar compositions of the key nutrients of concern, outlined in what follows. The Irish food pyramid was used as the starting point to allow potential future applications of this work (dietary assessment and dietary advice) to be easily understood in the context of existing dietary guidelines. The purpose of using these newly derived subgroups rather than existing food groupings, within the NANS data set, was to allow the food groups to be created in a data-driven manner that accounts for nutrients of public health importance and demonstrates a pathway for any future researcher to adapt to future data sets. Specifically, the features (or input variables) chosen for the clustering were the 12 nutrients used in the previously validated nutrition quality index, the Nutrient Rich Foods Index (NRF9.3) (19): protein, fiber, vitamin A, vitamin C, vitamin E, calcium, iron, magnesium, potassium, saturated fat, added sugar, and sodium; that is, foods were clustered based on their similarity in relation to those nutrients. Food supplements and energy-free foods were not included in this analysis, because they had not been included in the original validation studies of the NRF9.3 (19). Variables were in units per 100 kcal and were *z*-standardized before clustering. *K*-means clustering requires that the number of clusters is chosen before clustering. Within this study the number of clusters was chosen by applying 24 different indices used to determine the number of clusters to the data and choosing the number of clusters that was most frequently proposed among the indices (20). For 2 of the groups (cereal, bread, potato, pasta, and rice; milk, yogurt, and cheese), the clustering solution arising from the most frequently proposed number of clusters led to some clusters containing only 2–3 foods. In these instances, the next most frequently chosen value for cluster number was used. The range of possible values assessed for cluster number was from 2 to 10 inclusive. Charrad et al. (20) provide details on the indices used. The values of the indices obtained for the vegetables, salad, and fruit group of the food pyramid have been provided as an example in **Supplemental Table 1**A; values for all groups are available on request. Foods that did not have a specific group in the food pyramid were not clustered, but considered as individual food groups: alcoholic beverages, nonalcoholic beverages, and miscellaneous foods such as sauces, dips, and dressings. The fats, spreads, and oils group was not further split using clustering given the small number of foods in this group (*n* = 36). **Table 1** presents the original groups in the food pyramid and the number of clusters identified within each group. In total there were 12 food groups identified through clustering and the 3 remaining groups aforementioned that do not appear in the food pyramid (Table 1). These 15 food groups were then used to derive generic meals as described next.

**FIGURE 2:**
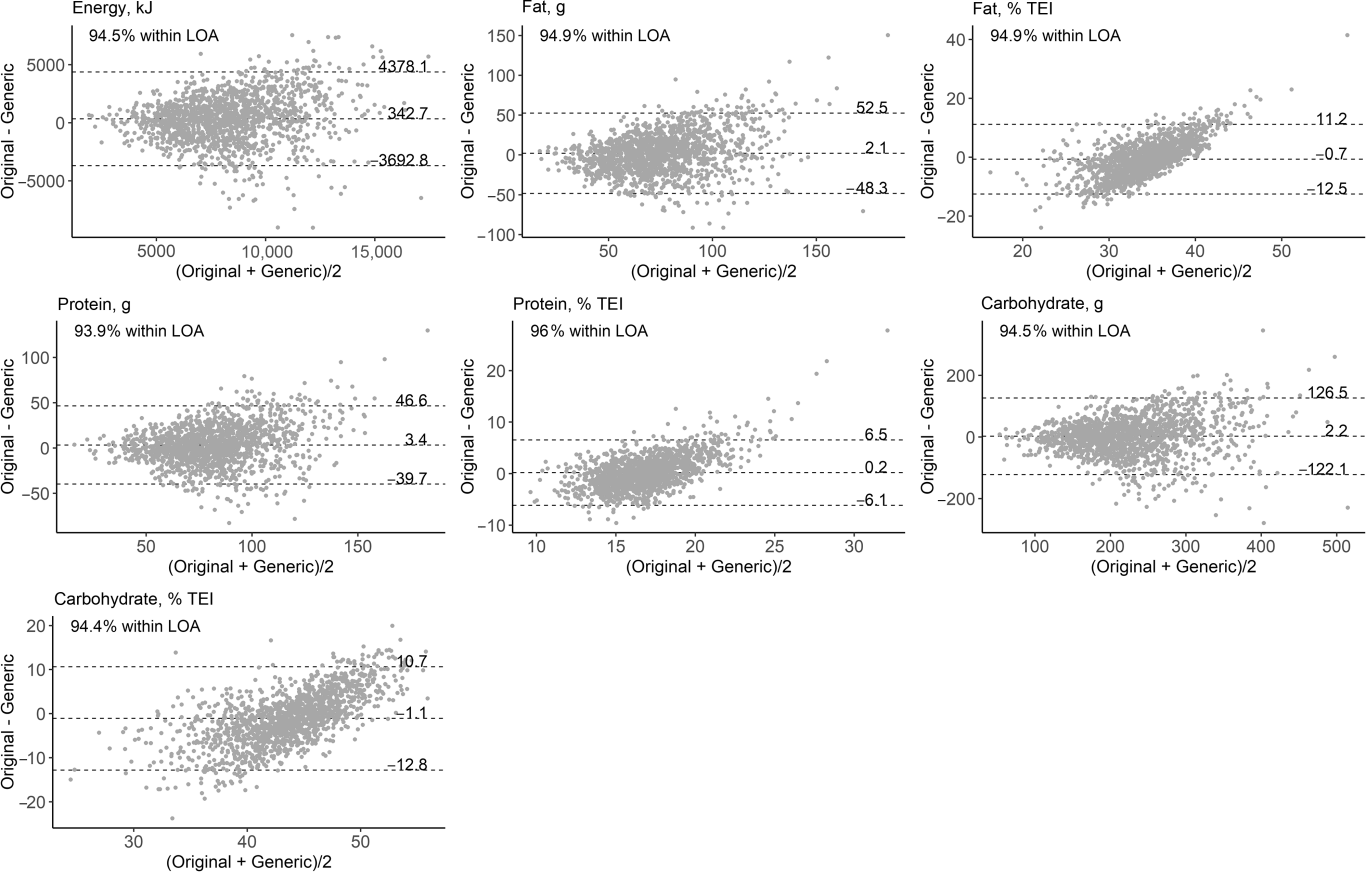
Bland–Altman plots for energy and macronutrients. The middle dashed line, and associated number, represents the mean difference in mean daily intakes between the original and generic databases. The upper and lower dashed lines, and associated numbers, represent the upper and lower LOA, respectively. Original and generic refer to the original and generic data sets. Each point represents an individual participant (*n* = 1500). LOA, limits of agreement; TEI, total energy intake.

**TABLE 1:** The numbers of clusters identified within each of the groups of the Irish Food Pyramid (food groups) with descriptions of the clusters^1^

Food groups	Clusters/groups, *n*	Description of clusters/groups
Vegetables, salad, and fruit	2	No clear distinction of traditional food groups or descriptions was observed between these clusters, simply called F&V1 and F&V2.
Cereal, bread, potato, pasta, and rice	3	Cereals Potatoes Breads, oats, pasta, and rice.
Milk, yogurt, and cheese	2	Milk and yogurt (and nondairy alternatives) Cheese.
Meat, poultry, fish, eggs, beans, and nuts	2	No clear distinction of traditional food groups or descriptions was observed between these clusters, called proteinfoods1 and proteinfoods2.
Fats, spreads, and oils (not further split)	1	Not applicable: only 1 group.
Foods and drinks high in fat, sugar, or salt	2	No clear distinction of traditional food groups or descriptions was observed between these clusters, simply called HFSS1 and HFSS2.
Other (not clustered, manually grouped)	3	Alcoholic beverages Nonalcoholic beverages Soups, sauces, and miscellaneous foods.

1 *K*-means clustering was carried out on the foods in each of the groups of the Irish food pyramid to identify clusters/subgroups within these groups. Clustering was performed separately for each group. Input variables for the clustering were the 12 nutrients in the Nutrient Rich Foods Index (NRF9.3) as reported by Fulgoni et al. (19). The number of clusters in each group was identified using 24 different indices to determine the optimal number of clusters between 2 and 10 based on the process described by Charrad et al. (20).

### Generic meals

After the *k*-means clustering to determine the 15 food groups, individual foods listed within each meal, reported by participants, were replaced with the food group to which they belonged. Individual meals were then defined by the individual food groups that made up each meal, meaning that for each food reported in a given meal, the food group to which it was assigned was used to represent that food, instead of the individual item (Figure 1). After this substitution of food groups in place of foods in the meal, an NRF9.3 (19) score was calculated for each individual meal as an indicator of nutritional quality. The score, developed by Fulgoni et al. (19), was calculated based on 9 nutrients to encourage (protein, dietary fiber, calcium, iron, potassium, magnesium, and vitamins A, C, and E) and 3 nutrients to limit (saturated fat, added sugar, and sodium). For each of these nutrients the quantity of the nutrient per 100 kcal present in each meal as a percentage of European reference intakes (RIs) was calculated. RIs were derived from Regulation (EU) No. 1169/2011 (21) where available. Those that were not available in that regulation (added sugar and dietary fiber) were instead derived from the European Food Safety Authority (EFSA) (22). RI percentages were limited to 100% to avoid overvaluing meals. The percentage RI scores for the nutrients to encourage were summed, as were the scores for the nutrients to limit. The nutrients to limit score was subtracted from the nutrients to encourage score to give an overall NRF9.3 score for each meal.

Within the NANS study participants self-selected the meal type to which each recorded intake belonged from the following list: breakfast, light meal (lunch), light meal (evening meal), main meal (lunch), main meal (evening meal), morning snack, afternoon snack, evening snack, night snack, alcoholic beverage, and nonalcoholic beverage. These meal types were condensed into the following 5 types: breakfasts, light meals, main meals, snacks, and beverages. Meals were also categorized by whether they were consumed on a weekday or a weekend day, giving rise to meals being divided into 10 categories (weekend breakfasts, weekday breakfasts, etc.). Within each meal category, the individual meals (defined by the food groups that they contained) were then grouped/clustered using partitioning around the medoids (PAM) clustering allowing for clustering based on both numerical and categoric variables (23, 24). The variables used to cluster were the NRF9.3 score for each meal and 15 binary variables indicating for each food group whether it was present or absent in the meal. PAM clustering requires that the number of clusters is chosen before clustering. Similar to the first clustering step, the number of clusters was chosen by applying 24 different cluster number indices to the data and choosing the number of clusters that was most frequently proposed among the indices (20). Values obtained from the various indices for the clustering of weekend breakfast meals have been provided in Supplemental Table 1B as an example; values for all meal types are available on request. The range of possible values assessed for cluster number was from 4 to 15 inclusive. This clustering process gave rise to a total of 63 clusters, i.e., generic meals.

The nutrient content of a given generic meal was then calculated as the mean nutrient content per 100 g of the individual meals that make up that generic meal. Before calculating mean nutrient content and portion size (described in what follows), meals considered to be outliers (based on energy and/or micronutrient content) within each generic meal were removed in a 2-step process. Firstly, meals that contained >1.5 times the IQR for energy were removed. Secondly, meals that contained >10 times the mean for any micronutrient were removed.

The weights (g) for the individual meals in each generic meal were used to determine generic portion sizes. Each generic meal was assigned 7 generic portion sizes. These portion sizes were determined for a given generic meal by ordering each of the individual meals by weight and dividing the meals into septiles. The median weights of each septile were assigned as the generic portion sizes for that meal (Figure 1).

### Generic intakes

To create the generic data set, the nutrient intake values of meals consumed in the original data set were replaced with the appropriate generic meal composition based on the portion size consumed (Figure 1). Mean daily intakes of the nutrients of interest were calculated using both the original and generic data sets.

### Statistical analysis

All analysis was carried out using R version 4.0.3 (25) in the RStudio integrated development environment (version 1.3.1093) (26). Mean nutrient intakes arising from the original and generic data sets were compared using a paired *t* test. To account for the multiple testing of energy and 30 nutrients, a Bonferroni-adjusted α of 0.05/31 was considered for statistical significance, i.e., *P* values < 0.002 were considered statistically significant. The nutrients compared between the 2 data sets included fat, saturated fat, monounsaturated fat, polyunsaturated fat, protein, carbohydrate, total sugars, added sugars [all of which were compared both using gram amounts and in terms of percentage total energy intake (% TEI)], dietary fiber (g), calcium (mg), iron (mg), potassium (mg), phosphorus (mg), sodium (mg), total vitamin A (μg), retinol (μg), carotene (μg), vitamins C (mg), D (μg), E (mg), B-12 (μg), and folate (μg).

The correlation of nutrient intakes between the 2 data sets was examined using Spearman rank correlation coefficients. Nutrient intakes in both data sets were divided into quartiles. This allowed for the calculation of the proportion of individuals who remained in the same quartile in both data sets (exact agreement), the proportion who were classified in the same or adjacent quartiles (exact agreement + adjacent), the proportion who were classified 2 quartiles apart (disagreement), and the proportion who were classified 3 quartiles apart (extreme disagreement). Bland–Altman analysis was carried out whereby the mean difference between the 2 data sets and the limits of agreement (mean difference ± 1.96 SD) for each nutrient were calculated. It is expected that ≥95% of observations will fall within the limits of agreement for comparable methods (27).

Finally, participants were classified, separately for both data sets, according to nutrient-based dietary guidelines (28–30). For example, they were classified as to whether their nutrient intakes were low, adequate, or high according to those guidelines. The nutrients assessed included protein, carbohydrate, fat, monounsaturated fat, polyunsaturated fat, saturated fat, salt, dietary fiber, calcium, iron, folate, thiamin, riboflavin, and vitamins A, B-12, and C. The proportion of individuals who were classified into the same category in both data sets was calculated for each nutrient.

## Results

Of the 1500 participants who completed the NANS study, 740 (49.3%) were male and 760 (50.7%) were female. The overall mean ± SD age was 44.5 ± 17 y. The overall mean ± SD BMI was 27.1 ± 5.0 kg/m^2^ (**Table 2**). Implausible estimates of energy intake were deemed to be those giving rise to an energy intake:basal metabolic rate ratio < 1.10 (31). The results of the analysis were not affected when participants with implausible estimates of energy intake were removed (data not shown). The results presented here, therefore, are those based on the data from all 1500 participants.

**TABLE 2:** Participant demographics

	Male	Female	Total
*n* (%)	740 (49.3)	760 (50.7)	1500 (100)
Age, y	43.8 ± 17.2	45.2 ± 16.8	44.5 ± 17.0
BMI, kg/m^2^	27.6 ± 4.6	26.6 ± 5.3	27.1 ± 5.0
Weight, kg	85.7 ± 14.7	69.7 ± 13.4	77.5 ± 16.1

A total of 27,336 individual meals were consumed by the 1500 participants during their 4-d recording period. Of these meals, 17,848 were consumed on a weekday and 9488 were consumed during the weekend. Overall, the most consumed meal type was snacks (*n* = 7710), followed by main meals (*n* = 6025), breakfast (*n* = 5698), light meals (*n* = 4455), and beverages (*n* = 3448). These individual meals were aggregated to 63 generic meals. These were comprised of 4 breakfasts, 8 light meals, 11 main meals, 4 snacks, and 4 beverages during the weekdays, and 4 breakfasts, 6 light meals, 14 main meals, 4 snacks, and 4 beverages during the weekend (Figure 1). **Supplemental Table 2** presents generic meal portion sizes and food-based descriptions based on the most frequently occurring foods in each food group of each meal. The nutritional content of each of the generic meals was determined from the mean nutritional content of the individual meals making up those generic meals excluding outlier meals. In total, 3825 meals were excluded on the basis of being outliers with regard to energy content and 2279 were excluded on the basis of being outliers with regard to micronutrient content. Therefore, a total of 21,232 meals were used to determine the nutrient content of the generic meals.

### Comparison of mean intakes

When comparing the sample population mean intakes of the original and the generic meal data (original data set compared with data set with generic meals substituted in), the mean percentage difference between estimated mean daily nutrient intakes for the original and generic data sets was 5.6%. The percentage difference between the data sets for all of the macronutrients and energy was <5%, ranging from 0.0% for total sugars as % TEI to 4.2% for added sugars as % TEI. The percentage differences for the micronutrients ranged from 3.0% for sodium (mg) to 25.3% for retinol (μg) (**Table 3**).

**TABLE 3:** Mean daily nutrient intakes estimated using the original food-based data set and the generic meal–based data set^1^

	Data set					
	Original, mean ± SD	Generic, mean ± SD	Difference, %	*P* value^2^	Effect size, Cohen's *d*	Effect size,^3^ magnitude
Energy, kJ	8431 ± 2747	8088 ± 2394	−4.1	<0.001*	0.166	Negligible
Fat, g	75.7 ± 29.4	73.6 ± 22.4	−2.8	0.002	0.081	Negligible
Fat, % TEI	33.8 ± 6.5	34.5 ± 2.9	2.1	<0.001*	0.108	Negligible
Saturated fat, g	29.7 ± 12.9	29.3 ± 9.4	−1.3	0.174	0.035	Negligible
Saturated fat, % TEI	13.3 ± 3.6	13.7 ± 1.4	3.0	<0.001*	0.135	Negligible
Monounsaturated fat, g	27.7 ± 11.4	26.7 ± 8.1	−3.6	<0.001*	0.094	Negligible
Monounsaturated fat, % TEI	12.3 ± 2.7	12.5 ± 1.1	1.6	0.003	0.077	Negligible
Polyunsaturated fat, g	13.3 ± 6.5	12.8 ± 3.9	−3.8	0.001*	0.088	Negligible
Polyunsaturated fat, % TEI	6.0 ± 2.2	6.0 ± 0.6	0.0	0.941	0.002	Negligible
Protein, g	83.3 ± 26.9	79.9 ± 22.0	−4.1	<0.001*	0.156	Negligible
Protein, % TEI	17.0 ± 3.6	16.8 ± 1.9	−1.2	0.020	0.060	Negligible
Carbohydrate, g	228 ± 78.9	226 ± 70.1	−1.0	0.181	0.035	Negligible
Carbohydrate, % TEI	42.9 ± 6.9	44.0 ± 3.5	2.6	<0.001*	0.179	Negligible
Total sugars, g	90.3 ± 43.1	87.7 ± 34.0	−2.9	0.006	0.072	Negligible
Total sugars, % TEI	16.9 ± 5.8	16.9 ± 3.1	0.0	0.765	0.008	Negligible
Added sugars, g	39.5 ± 31.1	39.2 ± 20.4	−0.8	0.573	0.015	Negligible
Added sugars, % TEI	7.2 ± 4.7	7.5 ± 2.5	4.2	0.002	0.080	Negligible
Dietary fiber, g	19.1 ± 7.9	19.1 ± 6.0	0.0	0.916	0.003	Negligible
Calcium, mg	895 ± 369	844 ± 256	−5.7	<0.001*	0.169	Negligible
Iron, mg	11.9 ± 5.0	11.3 ± 3.7	−5.0	<0.001*	0.145	Negligible
Potassium, mg	3035 ± 966	2859 ± 813	−5.8	<0.001*	0.262	Small
Phosphorus, mg	1378 ± 461	1297 ± 361	−5.9	<0.001*	0.236	Small
Sodium, mg	2493 ± 901	2418 ± 712	−3.0	<0.001*	0.096	Negligible
Total vitamin A, μg	1023 ± 831	875 ± 326	−14.5	<0.001*	0.191	Negligible
Retinol, μg	410 ± 623	307 ± 102	−25.3	<0.001*	0.167	Negligible
Carotene, μg	3674 ± 3174	3408 ± 1623	−7.3	<0.001*	0.095	Negligible
Vitamin C, mg	79.4 ± 52.4	71.9 ± 28.6	−9.4	<0.001*	0.178	Negligible
Vitamin D, μg	3.2 ± 2.6	2.7 ± 0.9	−15.6	<0.001*	0.229	Small
Vitamin E, mg	9.5 ± 4.9	8.9 ± 2.9	−6.3	<0.001*	0.116	Negligible
Total folate, μg	318 ± 152	285 ± 90.4	−10.3	<0.001*	0.255	Small
Vitamin B-12, μg	4.7 ± 3.5	3.8 ± 1.1	−19.1	<0.001*	0.265	Small

1 TEI, total energy intake.

2 *P* values were calculated using a paired *t* test. *Statistically significant using paired *t* test with Bonferroni-adjustment, *P *< 0.002.

3 Effect sizes < 0.2 were deemed to be negligible, and those ≥0.2 and <0.5 were deemed to be small (77).

Despite the small percentage differences for most nutrients, statistically significant differences were observed in mean intakes of 20 of the 30 nutrients assessed and for energy (adjusted *P* < 0.002). Nutrients that were not significantly different were fat in grams, saturated fat in grams, monounsaturated fat as %TEI, polyunsaturated fat as % TEI, protein as % TEI, carbohydrate in grams, total sugars in grams, total sugars as % TEI, added sugars in grams, added sugars as % TEI, and dietary fiber in grams (Table 3). All of the micronutrients assessed were found to differ significantly between the 2 data sets. However, of the 20 significant differences, 15 had a negligible effect size (Cohen's *d* < 0.2) and the remainder had a small effect size (Cohen's *d* ≥ 0.2 and <0.5). Those with a small effect size were potassium (Cohen's *d* = 0.262), phosphorus (Cohen's *d* = 0.263), vitamin D (Cohen's *d* = 0.229), folate (Cohen's *d* = 0.255), and vitamin B-12 (Cohen's *d* = 0.265) (Table 3).

Spearman rank correlation coefficients between the original and generic data sets ranged from 0.23 for polyunsaturated fat (% TEI) to 0.75 for potassium (mg), with a mean correlation across all nutrients of 0.53 (**Table 4**). Bland–Altman analysis identified 13 nutrients for which ≥95% of participants fell within the limits of agreement. The proportion of individuals that fell within the limits of agreement ranged from 93.7% for sodium (mg) to 98.4% for retinol (μg) (Table 4). **Figure 2** shows the Bland–Altman plots for the macronutrients.

**TABLE 4:** Correlation coefficients, cross-classification of quartiles, and percentage of participants within the Bland–Altman LOA between the original food-based data set and generic meal–based data set^1^

	Correlation (Spearman)^2^	Exact agreement,^3^ %	Exact agreement + adjacent,^4^ %	Disagreement,^5^ %	Extreme disagreement,^6^ %	Proportion within Bland–Altman LOA,^7^ %
Energy, kJ	0.69	52	89	10	1	94.5
Fat, g	0.54	43	82	14	3	94.9
Fat, % TEI	0.36	34	75	19	6	94.9
Saturated fat, g	0.51	41	81	15	4	95.3*
Saturated fat, % TEI	0.39	35	76	20	5	95.8*
Monounsaturated fat, g	0.52	42	81	15	3	94.8
Monounsaturated fat, % TEI	0.31	33	72	21	6	95.1*
Polyunsaturated fat, g	0.48	40	79	17	4	95.4*
Polyunsaturated fat, % TEI	0.23	31	70	22	8	96.6*
Protein, g	0.61	46	85	12	2	93.9
Protein, % TEI	0.46	39	79	17	4	96.0*
Carbohydrate, g	0.66	47	87	11	2	94.5
Carbohydrate, % TEI	0.46	39	79	17	4	94.4
Total sugars, g	0.64	44	86	12	2	94.5
Total sugars, % TEI	0.47	40	80	15	4	95.1*
Added sugars, g	0.61	46	84	14	2	94.3
Added sugars, % TEI	0.57	45	83	14	3	94.7
Dietary fiber, g	0.66	46	87	11	2	94.3
Calcium, mg	0.61	45	86	11	2	94.3
Iron, mg	0.62	46	86	12	2	94.4
Potassium, mg	0.75	53	91	8	1	94.9
Phosphorus, mg	0.69	48	88	10	1	94.1
Sodium, mg	0.56	42	82	15	3	93.7
Total vitamin A, μg	0.54	42	82	15	2	97.5*
Retinol, μg	0.47	37	79	17	4	98.4*
Carotene, μg	0.51	40	81	16	3	95.0*
Vitamin C, mg	0.62	43	86	12	2	94.2
Vitamin D, μg	0.34	33	74	21	6	96.0*
Vitamin E, mg	0.51	39	82	14	4	95.0*
Total folate, μg	0.61	46	85	13	2	94.9
Vitamin B-12, μg	0.44	38	79	16	5	97.5*

1 LOA, limits of agreement; TEI, total energy intake.

2 All correlations were statistically significant, *P* < 0.001.

3 Percentage of participants cross-classified into the same quartile.

4 Percentage of participants cross-classified into the same or adjacent quartiles.

5 Percentage of participants cross-classified 2 quartiles apart.

6 Percentage of participants cross-classified 3 quartiles apart.

7 The Bland–Altman LOA is the mean difference between the 2 data sets ± 1.96 SD. *Percentage within Bland–Altman LOA ≥95%.

### Individual intake classification

The proportion of participants remaining in the same quartile in both the original and generic data sets ranged from 31% for polyunsaturated fat (% TEI) to 53% for potassium (mg). Extreme disagreement of ≥5% of participants was observed for 6 nutrients: polyunsaturated fat as % TEI (8%), monounsaturated fat as % TEI (6%), total fat as % TEI (6%), vitamin D (6%), saturated fat as % TEI (5%), and vitamin B-12 (5%). The mean proportion of individuals in extreme disagreement across all nutrients was 3.3% (Table 4).

When participants were classified (high, adequate, or low) according to nutrient-based guidelines, the proportion of individuals who were classified into the same category ranged from 55.3% for polyunsaturated fat (% TEI) to 91.5% for both protein (g/kg body weight) and salt (g). The mean proportion of exact agreement across all nutrients was 79.8% (**Table 5**).

**TABLE 5:** Exact agreement between the original food-based data set and generic meal–based data set for categorization of participants’ nutrient intakes according to nutrient-based dietary guidelines^1^

Nutrient	Possible categories for classification of individual nutrient intakes	Proportion classified to the same category, %
Protein, g/kg BW	Low, adequate, and high	91.5
Carbohydrate, % TEI	Low, adequate, and high	65.5
Fat, % TEI	Low, adequate, and high	62.4
Monounsaturated fat, % TEI	Low, adequate, and high	85.5
Polyunsaturated fat, % TEI	Low, adequate, and high	55.3
Saturated fat, % TEI	Adequate and high	82.9
Salt, g	Adequate and high	91.5
Dietary fiber, g	Low and adequate	87.1
Calcium, mg	Low, adequate, and high	72.2
Iron, mg	Low, adequate, and high	90.2
Vitamin A, μg	Low, adequate, and high	76.8
Folate, μg	Low, adequate, and high	72.1
Thiamin, mg	Low and adequate	91.1
Riboflavin, mg	Low and adequate	90.9
Vitamin B-12, μg	Low and adequate	88.7
Vitamin C, mg	Low, adequate, and high	72.7

1 Participants were placed in categories according to nutrient-based guidelines. The percentages give the percentage of participants who were placed in the same category according to both the original and generic data sets. BW, body weight; TEI, total energy intake.

## Discussion

This study examined a novel generic meal–based method of exploratory analysis of dietary intake data. Unlike previous work in this area (7–11), the research described here utilizes data-driven methods of defining food groups based on nutrients from the NRF9.3 (19), and incorporates a range of standard portion sizes, which aimed to improve the accuracy of a meal-based intake method to estimate intakes at both a population and an individual level, which hitherto had not been considered. Differences between mean nutrient intakes determined using this generic meal–based method and the standard food-based method were found to be small or negligible. This study also compared nutrient intakes at the individual level, for the first time, demonstrating that participants can be classified according to nutrient-based dietary guidelines (for example, high, adequate, or low intakes) using the generic meal–based method, suggesting a possible role for the use of generic meals in meal-based dietary intake assessment.

Food groupings vary from study to study and are often tailored to the specific research in which they are used (32). Previous research examining generic meals has used pre-existing food groups as part of the process (7–11). The current research aimed to develop a generic meals process to assess dietary intakes with regard to nutrients of public health importance, specifically the 12 nutrients from the NRF9.3 (19). Because it was not feasible to group hundreds of foods manually based on their similarities for 12 different nutrients, clustering was applied instead. Although the use of nutrients in the derivation of generic meals appeared to improve the process, the use of the specific 12 nutrients in the generic meal–based approach may have affected the agreement between the 2 methods for intakes of those nutrients, above others. For example, the mean correlation between the methods for those nutrients used as input for *k*-means clustering was 0.57, whereas the mean correlation for nutrients that were not included was 0.48. This may provide scope for the inclusion of a different set of nutrients or other food components depending on the research question at hand. Indeed, previous studies that have clustered foods have also incorporated prior knowledge of nutrition in the selection of variables. For example, Pennington and Fisher (33) clustered fruit and vegetables using food components known to be primarily provided by these foods; Burden et al. (34) clustered foods based on macronutrient content with the aim of minimizing their differences between groups; and in a study concerning the antioxidant content of fruit and vegetables, Patras et al. (35) clustered foods based on their content of known antioxidant compounds.

In the present study, the choice of input variables for clustering deliberately focused on nutrients of known public health importance, introducing a degree of prior knowledge to a largely data-driven method. One of the limitations of this approach is that some of the variables included may be irrelevant or redundant for the purpose of clustering and thereby mask the underlying structures in the data (36). The alternative is to exclude prior knowledge and use a purely data-driven approach to selecting input variables for clustering (36, 37) that would enable foods to be grouped based only on the food components that differentiate those foods. Although these data-driven methods are valuable approaches to identify inherent patterns or groupings in the data, they may not perform as well in identifying groupings that are relevant to disease prevention or health promotion (14), which is of importance in this current study. Although a purely data-driven approach to clustering was not used in this instance, such approaches warrant further investigation because groupings driven by the underlying data structures of food composition may help researchers develop food groups for FFQs (33, 34), be useful in teaching about food composition, and aid health professionals to produce dietary advice about foods with similar compositions when the contents of multiple nutrients are of interest (33).

The methods described in this study build on previously published studies that used the frequent itemset data mining method (7–9) to derive the generic meals used in their analysis. These studies did not include portion size in their generic meal framework and the methods used accounted for food group “descriptions” but not nutrient content when identifying generic meals. The clustering method used in this current work derived generic meals based on nutrients of public health interest from the NRF9.3 (19) while also accounting for the food groups of which those generic meals were comprised. By identifying generic meals separately for weekdays and weekends, this method accounts for the differences that exist between weekday and weekend dietary intakes in relation to energy (38–40) and nutrient intake, food group consumption (38), and meal patterns (8). This approach was used to enhance the accuracy of the method, but also to ensure that identification of generic meals was guided by nutrients that are of public health importance and that are therefore central to dietary guidelines (41–43). As such, any dietary assessment or feedback tool incorporating this generic meal approach will be of relevance to existing guidelines.

Previous studies on generic meals have not incorporated portion size, and this is evident in the small variance in nutrient intakes seen in those studies (7, 9). The inclusion of portion size in the current study identified a greater range of nutrient intakes more reflective of the actual range of intakes observed in the original data set. The approach used here assigned 7 standard portion sizes to each generic meal based on actual meal intake weights. Comparable approaches have been used in established methods of dietary intake assessment.

Standard portion sizes used in semiquantitative FFQs are based on known population consumption patterns (44, 45), and participants must choose the portion size that they typically consume for each food in the questionnaire (46, 47). Whereas 24-h recalls have traditionally asked participants to estimate portion size using weight, household measures, or food models/images (48), Web-based versions also provide the option of standard portion sizes when weight/volume is unknown (49–53). The portion sizes presented for each food are based on various centiles of intake observed in national diet surveys (49–53). The majority of FFQs are designed to include portion size (54, 55). Despite this, it has been questioned whether this practice is warranted (54–56) given that improvements in estimates of nutrient intakes due to portion size are small (57, 58). During the development of the generic meals method described in the present study, both the inclusion and exclusion of portion size were assessed (data not shown). The inclusion of portion size resulted in better agreement with the original data for absolute nutrient intakes but had no impact on agreement for energy-adjusted nutrient intakes.

The percentage differences for most nutrients between the generic and original data for mean daily nutrient intakes (mean difference: 5.6%) were small considering the error that is inherent in dietary intake assessment. For comparison, mean daily estimated energy and protein intake using 4-d food records, three 24-h recalls, or an FFQ differs from doubly labeled water energy values by 20%–27% and from protein intake based on urinary nitrogen by 4%–10% (59). On comparing the results of the current study with previous studies of generic meals in the same population (7), improved agreement with the original data was observed for absolute nutrient intakes but similar for energy-adjusted intakes. The results of the current study, however, found poorer agreement than those reported for generic meals in a Japanese population (9). However, that study only reported energy-adjusted nutrient intakes and not absolute intakes. The differences between the current study and others, however, are not limited to portion size, and therefore the varying results may only be partly attributable to the inclusion of portion size in our study.

The current study demonstrated the ability of our generic meals approach to rank individuals within the sample population based on nutrient intakes. When individuals were classified into quartiles based on nutrient intakes using the generic data, the proportions classified in the same or adjacent quartiles as the original data were comparable with the equivalent proportions reported in previous generic meal research (72%–88%) (7) and in validation studies comparing FFQs with diet records (65%–88%) (60, 61) and 24-h recalls (55%–86%) (62, 63). This study also shows that participants can be classified according to nutrient-based guidelines using the generic data set, with the agreement with the original data set ranging from 55.3% to 91.5%. Several factors appear to have led to the range of agreements observed. Those nutrients with poorer agreement tended to be those expressed in % TEI, those that were not used as input variables in the clustering, and those that had 3 possible categories in the nutrient-based guidelines as opposed to 2. Further work is warranted to determine the best combination of these variables or whether this approach is limited owing to being unsuitable for certain nutrients.

The work presented here has significant potential for use in the provision of population and individual dietary advice. Currently, dietary advice is provided at a variety of levels. For example, government dietary guidelines are typically food and nutrient based, and targeted at national populations or certain population subgroups based on characteristics such as sex and age (64). Researchers have also shown that dietary advice can be targeted to population subgroups based on their metabolic profiles (65–67) and genetic profiles (67–69), and research has also examined the targeting of advice at an individual level based on existing dietary intakes (12, 70–74). Those studies providing advice at the individual level use food-based dietary assessment to classify individuals according to nutrient-based guidelines (e.g., high, adequate, or low). These classifications determine the advice provided (12, 70–74). The current study demonstrated for the first time the ability of a generic meals approach to classify individuals according to nutrient-based dietary guidelines. The results show promise for the use of generic meals in dietary assessment and feedback, for example, it may be possible for images of the generic meals described in this study to be presented to participants who could select the images of the meals and portions that most represent their dietary intakes, which in turn could be used to derive nutrient intakes, rather than recording each individual food consumed. Such a meal-based approach is likely to be more intuitive and less burdensome for end users (1, 6).

However, although the current study has described a method for the exploratory analysis of dietary intake data, this generic meal–based method has not yet been validated for use in a dietary intake assessment tool. The results presented here compare generic meal–based intakes with the food-based intakes from which those generic meals were derived. Given the inherent correlations between these data sets, this may overestimate the agreement between the 2 methods. The generic meal–based method described here may facilitate the development of a dietary assessment tool that is meal- rather than food-based. Further work is required to examine the generic meal–based method for use in collecting new dietary data from individuals and categorizing their intakes according to nutrient-based dietary guidelines. It is also unknown in meal-based research whether certain generic meals better represent their food-based equivalents than others because analysis of accuracy is carried out at the mean daily intake level (7–9). Analysis at the individual generic meal level could provide additional insights to further develop this process.

The work presented here should be considered in the context of its strengths and limitations. The primarily data-driven nature of the method limits the applicability of the results to other populations. However, the method itself can be applied to data sets from other populations to assess generic meal intakes. The nationally representative nature of the sample used here (16) strengthens the generalizability of the results within the Irish population. Despite this, the data used in this study were collected between 2008 and 2010, so the generic meals described may not necessarily be reflective of present-day meal intakes in Ireland. All self-reported dietary intake data are subject to measurement error (75). The use of a 4-d weighed dietary intake record to gather the dietary data used in this study will mitigate, but not eliminate, this problem (59, 76).

In conclusion, the novel generic meals approach described here characterizes the meals consumed in a nationally representative Irish population, providing a means to estimate nutrient intakes based on meal rather than food intake at the sample population and individual levels. Future work will aim to determine the utility of this exploratory work in other data sets and assess the adequacy of this approach in the development of a meal-based approach to dietary assessment. This may enable a meal-based method of dietary assessment that is less burdensome and more intuitive for individuals to complete than food-based methods (1, 6).

## Supplementary Material

nxac151_Supplemental_File

## Notes

Supported by Science Foundation Ireland Insight II grant 12/RC/2289_P2. The original collection of the data that were used in this work was supported by the Irish Department of Agriculture, Food and Marine and the Health Research Board under their joint Food for Health Research Initiative (2007–2012, FHRIUCC2). The funders had no role in the design, analysis, or writing of this article.

Author disclosures: The authors report no conflicts of interest.

Supplemental Tables 1 and 2 are available from the “Supplementary data” link in the online posting of the article and from the same link in the online table of contents at https://academic.oup.com/jn/.

Abbreviations used: NANS, National Adult Nutrition Survey; NRF, Nutrient Rich Foods Index; PAM, partitioning around the medoids; RI, reference intake; TEI, total energy intake.
