# Supplementary material for: A Clustering Approach to Meal-Based Analysis of Dietary Intakes Applied to Population and Individual Data
Source: J Nutr. 2022 Jul 11;152(10):2297–308. doi: 10.1093/jn/nxac151 (PMC9535445; doi:10.1093/jn/nxac151)
Supplement: nxac151_Supplemental_File [file nxac151_supplemental_file.docx]

Supplementary Table 1a: The values from the 24 indices used to determine the number of clusters when clustering the foods in the fruit and vegetables level of the Irish food pyramid, based on the R package from Charrad, et al. (1).^1^

| Index | Index Value for the Top 3 Cluster Numbers that were Deemed Optimal Most Frequently | | | Interpretation |
| --- | --- | --- | --- | --- |
|  | 2 | 5 | 10 |  |
| KL | 83.1* | 17.8 | 35.9 | Max value |
| CH | 148.4* | 92.9 | 82.5 | Max value |
| Hartigan | 31.2 | 17.2 | 9.0* | Max difference between hierarchy levels of index |
| CCC | -3.8 | 2.1 | 21.4* | Max value |
| Scott | 551.3 | 2159.6 | 3909.0* | Max difference between hierarchy levels of index |
| Marriot | 4.02^E+29^ | 6.07^E+28^* | 4.23^E+27^ | Max value of second differences between levels of the index |
| TrCovW | 117175.8 | 69270.3 | 26588.9* | Max difference between hierarchy levels of index |
| TraceW | 3845.3 | 2765.5* | 1873.9 | Max value of second differences between levels of the index |
| Friedman | 7.8 | 18.6* | 28.7 | Max difference between hierarchy levels of index |
| Rubin | 1.3 | 1.9* | 2.8 | Min value of second differences between levels |
| Cindex | 0.14 | 0.11 | 0.13 | Min value |
| DB | 1.62 | 1.49 | 1.38* | Min value |
| Silhouette | 0.5* | 0.3 | 0.2 | Max value |
| Duda | 1.2* | 1.1 | 1.9 | Smallest number of clusters such that index > critical value |
| Beale | -1.5* | -0.6 | -3.7 | Number of clusters such that critical value ≥ alpha |
| Ratkowsky | 0.31* | 0.29 | 0.25 | Max value |
| Ball | 1922.7 | 553.1 | 187.4 | Max difference between hierarchy levels of index |
| Ptbiserial | 0.5* | 0.5 | 0.5 | Max value |
| McClain | 0.2* | 0.5 | 0.7 | Min value |
| Dunn | 0.0* | 0.0 | 0.0 | Max value |
| Hubert |  |  |  | Graphical method^2^ |
| SDindex | 2.27* | 2.94 | 3.29 | Min value |
| Dindex |  |  |  | Graphical method^2^ |
| SDbw | 1.5 | 1.7 | 1.7 | Min value |
| Total | 10 | 4 | 5 |  |

^1^Information on the interpretation of the various indices was adapted from Charrad, et al. (1).

The optimal number of clusters as chosen by each index is indicated by an asterisk. The number of clusters chosen for the subsequent clustering step was the cluster number that was deemed to be optimal most frequently by the various indices. The range of cluster numbers that was assessed was 2–10.

^2^The two graphical methods used involved choosing the cluster number where a peak occurred on the second differences plot for those indices.

Supplementary Table 1b: The values from the 24 indices used to determine the number of clusters when clustering the meals consumed at breakfast on weekends, based on the R package from Charrad, et al. (1).^1^

| Index | Index Value for the Top 3 Cluster Numbers that were Deemed Optimal Most Frequently | | | Interpretation |
| --- | --- | --- | --- | --- |
|  | 4 | 8 | 12 |  |
| KL | 1.27 | 0.78 | 1.07 | Max value |
| CH | 0.21 | 2.97* | 2.02 | Max value |
| Hartigan | 1.0 | 0.0* | 0.2 | Max difference between hierarchy levels of index |
| CCC | -243.8* | -472.6 | -563.4 | Max value |
| Scott | 5900.9 | 7532.8 | 9252.3* | Max difference between hierarchy levels of index |
| Marriot | 4.64^E+37^ | 8.34^E+37^ | 8.07^E+37^* | Max value of second differences between levels of the index |
| TrCovW | 3.60^E+11^ | 3.53^E+11^ | 3.53^E+11^ | Max difference between hierarchy levels of index |
| TraceW | 2.40^E+06^ | 2.38^E+06^ | 2.38^E+06^ | Max value of second differences between levels of the index |
| Friedman | 20.6 | 21.9 | 23.2* | Max difference between hierarchy levels of index |
| Rubin | 2.38 | 2.40* | 2.40 | Min value of second differences between levels |
| Cindex | 0.23 | 0.22 | 0.22 | Min value |
| DB | 2.87 | 3.48 | 5.20 | Min value |
| Silhouette | 0.09* | 0.02 | -0.03 | Max value |
| Duda | 1.0* | 1.6 | 1.0 | Smallest number of clusters such that index > critical value |
| Beale | 0.0* | -2.6 | 0.0 | Number of clusters such that critical value ≥ alpha |
| Ratkowsky | 0.031 | 0.075 | 0.076* | Max value |
| Ball | 6.01^E+05^ | 2.97^E+05^ | 1.98^E+05^ | Max difference between hierarchy levels of index |
| Ptbiserial | 0.05 | 0.23 | 0.25 | Max value |
| McClain | 0.002* | 0.015 | 0.018 | Min value |
| Dunn | 0.17* | 0.10 | 0.10 | Max value |
| Hubert |  |  |  | Graphical method^2^ |
| SDindex | 1.43* | 1.80 | 2.34 | Min value |
| Dindex |  | * |  | Graphical method^2^ |
| SDbw | 1.74 | 1.51 | 0.68* | Min value |
| Total | 7 | 4 | 5 |  |

^1^Information on the interpretation of the various indices was adapted from Charrad, et al. (1). The optimal number of clusters as chosen by each index is indicated by an asterisk. The number of clusters most frequently deemed to be optimal by the various indices was chosen for the subsequent clustering step. The range of cluster numbers that was assessed was 4–15.

^2^The two graphical methods used involved choosing the cluster number where a peak occurred on the second differences plot for those indices.

Supplementary Table 2: Food-based descriptions of generic meals and their portion sizes.

| **Time of Week** | **Meal Type** | **Food-Based Description** | **Portion Sizes in Grams** | | | | | | |
| --- | --- | --- | --- | --- | --- | --- | --- | --- | --- |
|  |  |  | **1** | **2** | **3** | **4** | **5** | **6** | **7** |
| Weekend | Breakfast | Bread/toast with fat spread, tea with milk, eggs, sausages, rashers, and pudding | 275 | 372 | 440 | 515 | 600 | 747 | 1040 |
| Weekend | Breakfast | Bread/toast, with fat spread and marmalade/jam. Tea with milk and sugar. | 244 | 338 | 391 | 480 | 554 | 678 | 939 |
| Weekend | Breakfast | Bread/porridge, tea with milk, and fruit juice/whole fruit | 214 | 379 | 473 | 568 | 676 | 845 | 1135 |
| Weekend | Breakfast | Cereal with tea and milk | 151 | 252 | 334 | 425 | 499 | 594 | 831 |
| Weekday | Breakfast | Toast or porridge | 74 | 137 | 200 | 259 | 356 | 462 | 695 |
| Weekday | Breakfast | Bread/toast with fat spread and jam/marmalade, tea with milk and sugar. | 270 | 340 | 391 | 458 | 549 | 649 | 911 |
| Weekday | Breakfast | Bread or porridge, tea with milk, and fruit juice/whole fruit | 350 | 457 | 542 | 619 | 715 | 882 | 1191 |
| Weekday | Breakfast | Cereal with milk and tea | 170 | 275 | 360 | 443 | 512 | 630 | 917 |
| Weekend | Light Meal | Sandwich with bread, mayonnaise/dressing, meat with veg and tea with milk | 422 | 509 | 574 | 631 | 709 | 846 | 1190 |
| Weekend | Light Meal | Sandwich with bread, fat spread, meat/eggs, tea and milk | 294 | 378 | 438 | 500 | 612 | 741 | 996 |
| Weekend | Light Meal | Bread and soup and fruit | 122 | 235 | 300 | 384 | 456 | 648 | 932 |
| Weekend | Light Meal | Sandwich with bread, cheese, ham, and tea | 157 | 296 | 392 | 441 | 534 | 715 | 951 |
| Weekend | Light Meal | Sandwich with fat spread and meat, with cookie/chocolate, tea, and milk | 334 | 406 | 463 | 528 | 627 | 745 | 959 |
| Weekend | Light Meal | Cookies/chocolate/cake with tea and milk | 236 | 318 | 406 | 486 | 581 | 713 | 1040 |
| Weekday | Light Meal | Sandwich with bread, fat spread, eggs, sausages, and bacon, with tea and milk and sugar. | 334 | 404 | 456 | 527 | 601 | 679 | 853 |
| Weekday | Light Meal | Bread, fat spread, with banana, and tea with milk. | 381 | 458 | 545 | 635 | 714 | 854 | 1133 |
| Weekday | Light Meal | Bread with chicken and water. | 221 | 350 | 438 | 544 | 640 | 757 | 949 |
| Weekday | Light Meal | Ham sandwich with tea and milk | 194 | 365 | 435 | 480 | 592 | 709 | 935 |
| Weekday | Light Meal | Bread with soup and/or whole fruit | 148 | 243 | 301 | 380 | 481 | 620 | 916 |
| Weekday | Light Meal | Sandwich with bread, mayonnaise/dressing, meat with veg and water | 242 | 417 | 508 | 588 | 734 | 853 | 1120 |
| Weekday | Light Meal | Sandwich with bread, cheese, fat spread, ham, with tea and milk | 300 | 400 | 449 | 515 | 615 | 710 | 930 |
| Weekday | Light Meal | Tea and milk with cookie/chocolate/cake. | 237 | 321 | 408 | 469 | 577 | 676 | 947 |
| Weekend | Main Meal | Potatoes with beef, veg, tea, milk, and sweet dessert. | 675 | 771 | 935 | 1062 | 1154 | 1269 | 1604 |
| Weekend | Main Meal | Bread/rice with chicken curry /stir fry | 358 | 518 | 668 | 795 | 933 | 1123 | 1620 |
| Weekend | Main Meal | Chips/potatoes with meat and tea | 433 | 508 | 607 | 678 | 772 | 943 | 1300 |
| Weekend | Main Meal | Potatoes, ham/beef, green veg, and water | 390 | 595 | 740 | 825 | 934 | 1109 | 1528 |
| Weekend | Main Meal | Potatoes, beef, gravy, carrots, and green veg. | 315 | 404 | 500 | 621 | 721 | 861 | 1132 |
| Weekend | Main Meal | Potatoes, beef, veg, gravy, water. | 481 | 637 | 756 | 909 | 1073 | 1241 | 1483 |
| Weekend | Main Meal | Potatoes with chicken, veg, and water | 451 | 651 | 765 | 867 | 975 | 1176 | 1347 |
| Weekend | Main Meal | Bread with fat spread, ham, egg, onions, coleslaw, tea and milk | 486 | 641 | 760 | 834 | 888 | 1007 | 1346 |
| Weekend | Main Meal | Potatoes/chips, beef, veg, sugar-sweetened beverage | 382 | 498 | 601 | 716 | 811 | 936 | 1124 |
| Weekend | Main Meal | Bread, rice/spaghetti, sauce, chicken, pulses, veg, and water | 328 | 578 | 713 | 817 | 960 | 1082 | 1613 |
| Weekend | Main Meal | Potatoes, beef/bacon/ham, vegetables, cookie, tea, and milk | 588 | 691 | 808 | 894 | 1010 | 1160 | 1439 |
| Weekend | Main Meal | Bread with meat, tea, milk, and cookies | 410 | 596 | 690 | 771 | 908 | 1108 | 1442 |
| Weekend | Main Meal | Rice/bread/pizza with beef/poultry dish. | 200 | 346 | 429 | 547 | 700 | 854 | 1298 |
| Weekend | Main Meal | Potatoes with peas and meat | 241 | 348 | 454 | 571 | 700 | 825 | 1006 |
| Weekday | Main Meal | Bread, beef dishes, tea, milk, and sugar | 411 | 533 | 630 | 740 | 895 | 1041 | 1358 |
| Weekday | Main Meal | Potatoes, meat/fish, and veg | 338 | 430 | 520 | 605 | 701 | 878 | 1165 |
| Weekday | Main Meal | Potatoes, chicken, peas | 264 | 382 | 452 | 571 | 656 | 790 | 1113 |
| Weekday | Main Meal | Rice/pasta/bread, meat products, beg, sauce, and water | 436 | 594 | 698 | 823 | 948 | 1083 | 1291 |
| Weekday | Main Meal | Rice/pasta, chicken dishes, water | 368 | 542 | 654 | 764 | 884 | 1025 | 1322 |
| Weekday | Main Meal | Rice/pasta/bread, chicken/fish, veg, tea, milk, sugar, chocolate. | 502 | 682 | 763 | 835 | 909 | 1036 | 1314 |
| Weekday | Main Meal | Potatoes, chicken/fish, veg, water, tea, milk. | 560 | 663 | 772 | 897 | 989 | 1097 | 1434 |
| Weekday | Main Meal | Rice/pasta/bread, beef/chicken | 210 | 347 | 437 | 525 | 657 | 777 | 1067 |
| Weekday | Main Meal | Potatoes, beef/ham, veg | 534 | 702 | 795 | 909 | 1011 | 1163 | 1430 |
| Weekday | Main Meal | Chips/potatoes, fish/beef/bacon/chicken, water | 353 | 527 | 629 | 716 | 840 | 979 | 1310 |
| Weekday | Main Meal | Potatoes, beef/fish/chicken | 375 | 565 | 657 | 782 | 901 | 1043 | 1374 |
| Weekend | Snack | Bread/scone with fat spread and tea with milk | 169 | 296 | 348 | 398 | 455 | 546 | 767 |
| Weekend | Snack | Cookie/chocolate with tea and milk | 202 | 252 | 283 | 312 | 345 | 403 | 632 |
| Weekend | Snack | Cookie/chocolate with water | 26 | 66 | 200 | 267 | 354 | 522 | 809 |
| Weekend | Snack | Piece of fruit | 49 | 113 | 160 | 250 | 359 | 480 | 790 |
| Weekday | Snack | Cookies/chocolate | 22 | 40 | 64 | 129 | 250 | 363 | 592 |
| Weekday | Snack | Bread with fat spread and tea and milk | 201 | 301 | 337 | 384 | 437 | 527 | 766 |
| Weekday | Snack | Cookies/chocolate and tea and milk and sugar | 176 | 247 | 277 | 307 | 340 | 393 | 601 |
| Weekday | Snack | Fruit and water | 97 | 140 | 200 | 275 | 372 | 519 | 761 |
| Weekend | Beverage | Spirits or lager with cola or red bull | 200 | 250 | 330 | 468 | 600 | 915 | 1125 |
| Weekend | Beverage | Lager/stout | 175 | 378 | 574 | 663 | 1144 | 1665 | 2272 |
| Weekend | Beverage | Milk and tea/coffee | 220 | 245 | 260 | 275 | 309 | 332 | 542 |
| Weekend | Beverage | Water | 160 | 250 | 284 | 366 | 568 | 628 | 1000 |
| Weekday | Beverage | Coffee/tea with milk and sugar | 211 | 255 | 272 | 284 | 308 | 330 | 470 |
| Weekday | Beverage | Water | 119 | 225 | 284 | 330 | 500 | 568 | 1000 |
| Weekday | Beverage | Tea and milk | 210 | 232 | 245 | 262 | 287 | 315 | 462 |
| Weekday | Beverage | Lager/stout | 145 | 375 | 568 | 638 | 990 | 1148 | 1722 |

**References**

1. Charrad M, Ghazzali N, Boiteau V, Niknafs A. NbClust: An R Package for Determining the Relevant Number of Clusters in a Data Set. Journal of Statistical Software 2014;61(6).
